# p35 is a Crucial Player in NK-cell Cytotoxicity and TGFβ-mediated NK-cell Dysfunction

**DOI:** 10.1158/2767-9764.CRC-22-0497

**Published:** 2023-05-05

**Authors:** Derek P. Wong, Claire E. Fritz, Daniel Feinberg, Alex Y. Huang, Reshmi Parameswaran

**Affiliations:** 1 Department of Pathology, Case Western Reserve University, Cleveland, Ohio.; 2 Pediatric Hematology and Oncology, The Angie Fowler Adolescent & Young Adult Cancer Institute, University Hospitals Rainbow Babies & Children's Hospital, Cleveland, Ohio.; 3 The Case Comprehensive Cancer Center, Case Western Reserve University School of Medicine, Cleveland, Ohio.; 4 Division of Hematology/Oncology, Department of Medicine, Case Western Reserve University, Cleveland, Ohio.

## Abstract

Natural killer (NK) cells are innate lymphocytes with cytotoxic activity. Understanding the factors regulating cytotoxicity is crucial for improving NK-cell adoptive therapies. Here, we studied a previously unknown role of p35 (CDK5R1), a coactivator of cyclin-dependent kinase 5 (CDK5) in NK-cell function. p35 expression was thought to be neuronal-specific and the majority of studies are still focused on neuronal cells. Here, we show that CDK5 and p35 are expressed in NK cells and are kinase-active. NK cells from p35 knockout mice were analyzed and showed significantly increased cytotoxicity against murine cancer cells, while they did not show any differences in cell numbers or maturation stages. We confirmed this using human NK cells transduced with p35 short hairpin RNA (shRNA), showing similar increase in cytotoxicity against human cancer cells. Overexpression of p35 in NK cells resulted in moderate decrease in cytotoxicity, while expressing a kinase-dead mutant of CDK5 displayed increased cytotoxicity. Together, these data suggest that p35 negatively regulates NK-cell cytotoxicity. Surprisingly, we found that TGFβ, a known negative regulator of NK-cell cytotoxicity, induces p35 expression in NK cells. NK cells cultured with TGFβ exhibit reduced cytotoxicity, while NK cells transduced with p35 shRNA or mutant CDK5 expression exhibited partial reversal of this inhibitory effect pointing to an interesting hypothesis that p35 plays an important role in TGFβ-mediated NK-cell exhaustion.

**Significance:**

This study reports a role for p35 in NK-cell cytotoxicity and this might help to improve NK-cell adoptive therapy.

## Introduction

Natural killer (NK) cells are cytotoxic lymphocytes that are part of the innate immune system and have critical roles in antiviral immunity and cancer immunosurveillance (1).They differ from B and T lymphocytes in that NK cells express a set of invariant activating receptors that can broadly recognize aberrant cells, such as cancer cells (2). Unlike T cell–based therapies, allogeneic NK-cell therapy demonstrates much lower risk of serious GVHD (3). These unique features make NK cells attractive candidates for cancer immunotherapy (4). However, NK cells are confronted with a number of clinical challenges, including decreased cytotoxicity and tumor avoidance of NK-cell surveillance (4). Elevated levels of TGFβ secretion have been observed in patients with cancer and have been implicated in NK-cell dysfunction and immune evasion in these patients (5–7). An improved understanding of the basic molecular pathways that govern NK cytotoxicity could significantly enhance NK cell–based immunotherapies.

**FIGURE 1:**
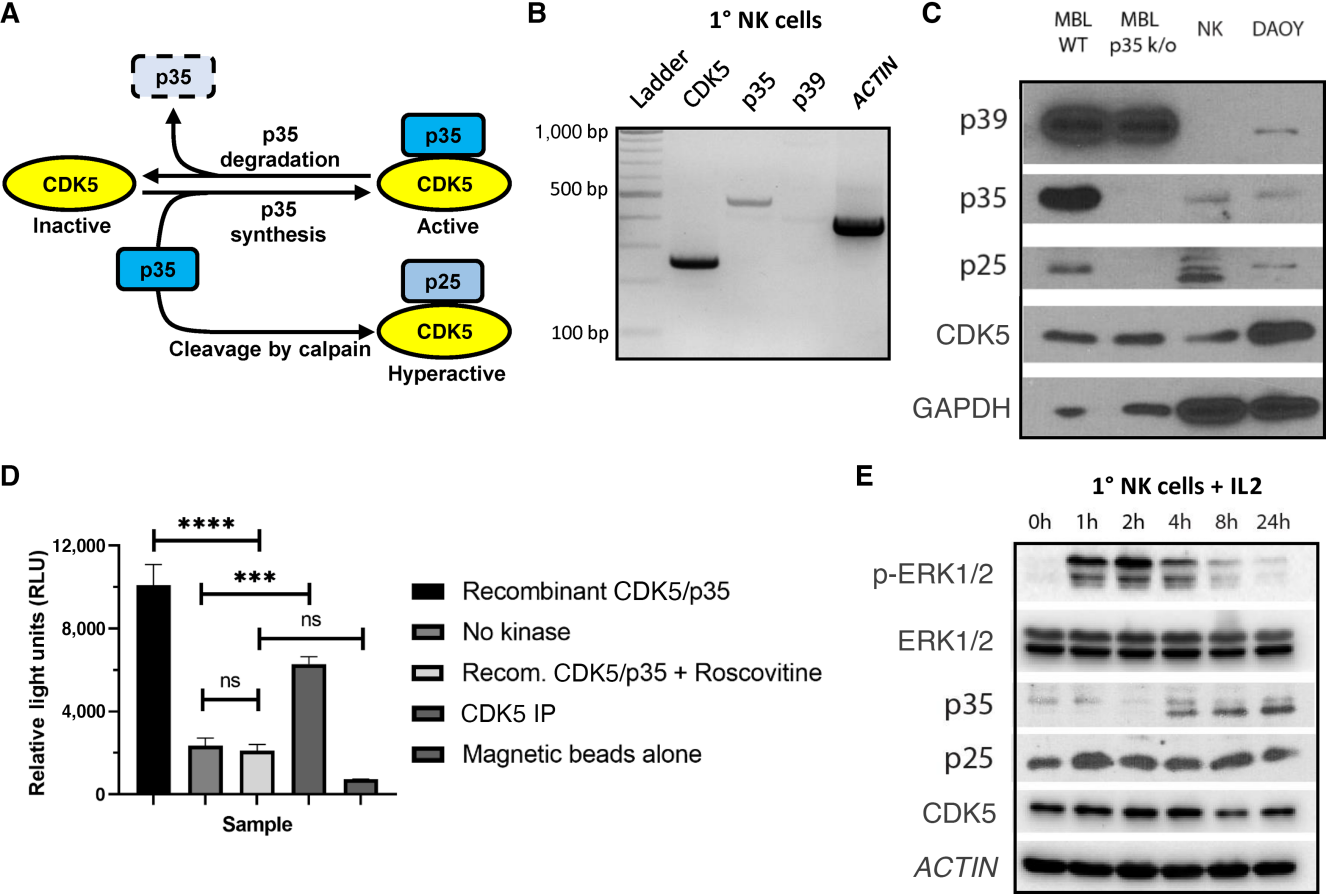
CDK5/p35 are expressed in NK cells and display kinase activity. **A,** Schematic of CDK5 kinase activity, which is primarily dependent on the binding of the coactivator p35 in non-neuronal cell types. **B,** RT-PCR was performed using RNA isolated from 1° NK cells to identify transcriptional expression of *CDK5*, *p35*, *p39*, and *ACTIN*. PCR products were run on a 2% agarose gel with a 100 bp DNA ladder as reference. **C,** Western blot analysis was used to identify translational expression of CDK5, p35, and p39 in 1° NK cells. MBL from WT mice and protein lysate from the DAOY human medulloblastoma cell line were used as positive controls of expression, while MBL from p35 k/o mice were used as a negative control of p35 expression. **D,** CDK5 kinase activity was measured using an assay in which CDK5 was mixed with ATP and the known CDK5 substrate histone H1. CDK5 from NK92 cells was immunoprecipitated (CDK5 IP) using polyclonal CDK5 antibody and protein A-magnetic beads. A total of 25 ng of recombinant CDK5/p35 provided in the kinase kit was used as a positive control of kinase activity, while the addition of 50 μmol/L of roscovitine was used as a negative control. No kinase and magnetic beads alone conditions were also used as negative controls. ns, not significant; ***, *P* < 0.001; ****, *P* < 0.0001. Graphs display mean ± SD, *n* = 3 technical replicates, one-way ANOVA with Tukey multiple comparisons test. **E,** 1° NK cells were cultured in cytokine-free media for 48 hours, followed by culture with 200 U/mL IL2. Protein lysate was collected at various timepoints.

Cyclin-dependent kinase 5 (CDK5) is a proline-directed serine/threonine kinase and a member of the CDK family (8). Similar to other CDK proteins, for which enzymatic activity is dependent on the association of a regulatory protein (e.g., CDK4/6 and CYCLIN D1), CDK5 kinase activity depends on the binding of the coactivator p35 (CDK5R1) or p39 (CDK5R2; ref. 8). p35 itself has a short half-life of approximately 20–30 minutes and is readily ubiquitinated and degraded by the proteasome (9). Thus, CDK5 kinase activity is finely regulated by the synthesis and degradation of p35 (Fig. 1A). Whereas CDK5 is present in all tissues, the coactivators p35 and p39 are only expressed in some cell types, such as neuronal cells, in which they were first discovered (10, 11). Other coactivators of CDK5 reported are CYCLIN I (CCNI) and its homolog CCNI2 (12, 13). Indeed, the vast majority of past and present reports on CDK5 have detailed its many roles in neuronal tissue and neurodegenerative diseases such as Alzheimer disease, in which it is believed that calpain-mediated cleavage of p35 to p25 leads to CDK5/p25 hyperactivity and phosphorylation of tau protein (14, 15). Compared with other known CDK cell-cycle regulators (e.g., CDK1, CDK2, CDK4/6), CDK5 plays a much more nuanced and uncertain role in the regulation of the cell cycle (16). However, over the past two decades, there have been many discoveries of p35 expression in non-neuronal cells and tissues that have revealed new and unexpected roles for CDK5 (17). For example, research has revealed diverse roles for CDK5 in immune cell types. In neutrophils, CDK5/p35 promotes granule secretion under stimulation by GTP (18). In macrophages, CDK5/p35 inhibits the production of IL10 during early-stage macrophage stimulation (19). In T cells, expression of both CDK5 and p35 are induced upon stimulation of the T-cell receptor, and CDK5 activity was found to be a requirement for T-cell activation (20). CDK5 activity is also important for IL2 gene expression (21). CDK5 function has also been found to be generally prosurvival in a number of different cancers, including breast cancer and medulloblastoma (22, 23). In fact, TGFβ was shown to induce CDK5 and p35 expression while promoting epithelial–mesenchymal transition in breast cancer cells (22). TGFβ has also been shown to induce p35 expression in fibroblasts and adipocytes (24). In NK cells, TGFβ exerts many immunosuppressive effects that inhibit their cytotoxicity, proliferation, and metabolic activity (25–27). TGFβ has been shown to reduce expression of activating receptors such as NKp30 (NCR3) and NKG2D (KLRK1), and TGFβ also inhibits NK-cell antibody-dependent cellular cytotoxicity and CD16-mediated IFNγ production (28, 29). Some mechanisms of TGFβ signaling in NK cells have been identified, such as the mTOR pathway (27). However, much remains to be investigated about how TGFβ mediates these pleiotropic effects in NK cells.

To date, there have not been any known reports that describe the expression or function of CDK5/p35 in NK cells. Our data reveal that CDK5 and p35 are both expressed and kinase active in NK cells. Using various genetic tools to modulate CDK5/p35 expression or activity, as well as using NK cells isolated from p35 knockout (p35 k/o) mice, we methodically demonstrate that p35 negatively regulates NK-cell cytotoxicity. Furthermore, we show that NK cells cultured with TGFβ induce the expression of p35, while TGFβ-mediated inhibition of NK-cell cytotoxicity can be partially reversed through the inhibition of p35 expression. An improved understanding of how TGFβ signaling and CDK5/p35 kinase activity intersect may lead to the discovery of novel methods for enhancing NK cell–based immunotherapies.

## Materials and Methods

### Cell Lines

Jeko-1 (catalog no. CRL-3006), NK92 (catalog no. CRL-2407), and HEK293T/17 (catalog no. CRL-11268) cells were purchased from the ATCC. Jeko-1 cells were cultured in RPMI1640 media (Sigma-Aldrich) supplemented with 10% FBS (Sigma-Aldrich) and 1% penicillin-streptomycin (P/S, Gibco). NK92 cells were cultured in αMEM media without ribonucleosides and deoxyribonucleosides (Sigma-Aldrich) supplemented with 12.5% FBS, 12.5% horse serum (Sigma-Aldrich), 1% P/S, 2 mmol/L l-glutamine (Gibco), 0.02 mmol/L folic acid (Acros Organics), 0.1 mmol/L inositol (Acros Organics), 0.1 mmol/L β-mercaptoethanol (55 μmol/L), and 1.5 g/L sodium bicarbonate (Thermo Fisher Scientific). For routine cell culture maintenance, NK92 cells were split every 2–3 days with fresh addition of 200 U/mL recombinant human IL2 (BioLegend). HEK293T cells were cultured in DMEM (Sigma-Aldrich) supplemented with 10% FBS and 1% P/S. DAOY cells were obtained from Alex Huang's lab and were cultured in DMEM (10% FBS, 1% P/S). B16F10 cells were obtained from Dr. Barbara Bedogni (University of Miama, Miami, FL) and were cultured in DMEM (10% FBS, 1% P/S). K562 feeder cells were obtained from Dr. Dean Lee (Nationwide Children's Hospital, Columbus, OH) and were cultured in RPMI (10% FBS, 1% P/S). Cell lines were authenticated using short tandem repeat DNA profiling (DDC Medical) with minimum 80% identity at profiled loci. Cell lines in culture were tested for *Mycoplasma* contamination every 4 months using Lookout *Mycoplasma* PCR Detection Kit (Sigma-Aldrich). Individual cryovials were thawed and used after 1–2 months in culture.

### Chemicals, Proteins, and Western and Flow Antibodies

EW-7197 was purchased from Selleck Chemicals. Roscovitine (Sigma-Aldrich) was solubilized in DMSO and stored in 10 mmol/L aliquots at −20°C. Human TGFβ was ordered from BioLegend. The following Western blot antibodies were purchased from Cell Signaling Technology: p44/42 MAPK (MAPK3/1, or ERK1/2) #9102; phospho-p44/42 MAPK (ERK1/2; Thr202/Tyr204) #9101; CDK5 (1H3) #12134; p35/p25 (C64B10) #2680; p39 #3275; phospho-SMAD2 (Ser465/467; 138D4) #3108; phospho-SMAD3 (Ser423/425; C25A9) #9520. The following Western blot antibodies were purchased from Santa Cruz Biotechnology: GAPDH (0411) #sc-47724 and β-ACTIN (ACTB) (C4) #sc-47778. All flow cytometry antibodies were purchased from BioLegend: CD11b (ITGAM)-FITC (M1/70); CD27-PE (LG.3A10); NK1.1 (KLRB1C)-APC (PK136); CD3 (CD3ε)-PerCP (145-2C11); NKG2D (KLRK1)-PE (CX5); 2B4 (CD244a)-FITC [m2B4 (B6)458.1]; LY6C-PE (HK1.4); LY49C/F/I/H (KLRA3/KLRA6/KLRA9/KLRA8)-PE (14B11); NKp46 (NCR1)-PE (29A1.4); NKG2A (KLRC1)-PE (16A11).

### Animals

p35 ± heterogeneous (p35 Het) mice were obtained from Alex Huang's lab and were bred from crossing wild-type (WT) C57BL/6 mice and the p35 k/o strain B6.129S4-*CDK5R1^tm1Lht^*/J (RRID: ISMR_JAX:004163), which was obtained originally from the Jackson Laboratory. Mice were housed in the pathogen-free animal facility at the Wolstein Research Building. Mouse euthanasia procedure was approved by the CWRU Institutional Animal Care and Use Committee and was conducted in accordance with their protocols.

### Murine NK-cell Analysis

Mouse spleen was processed via mechanical disruption and flow through a 70 μm cell strainer to obtain single-cell suspensions, while a syringe and 25-gauge needle were used to obtain bone marrow cells by flushing them through mouse femur and tibia with ice-cold PBS. Red blood cell lysis was performed using RBC Lysis Buffer (Thermo Fisher Scientific). Remaining cells were either stained for flow cytometry analysis, or murine NK cells were isolated for cytotoxicity assays using either the MojoSort Mouse NK Cell Isolation Kit (BioLegend) or the mouse NK Cell Isolation Kit from Miltenyi Biotec, both of which are negative isolation kits. For murine NK (mNK) cell quantification, splenocytes and bone marrow cells from the left femur and tibia were resuspended in 3 mL or 500 μL of PBS, respectively. A total of 50 μL of cell suspension were stained using anti-CD3 and anti-NK1.1 flow antibody, then mixed with 20 μL of Precision Count Beads (BioLegend) with a known bead concentration. The following formula was used to determine cell concentration, which was then multiplied by the total volume of suspension to obtain the total mNK cell count in the respective compartment: Cells/μL = [(cell count × beads volume)/(beads count × cell volume)] × beads concentration.

### Vectors

pLKO.1 lentiviral short hairpin RNA (shRNA) expression vector was obtained from Sigma-Aldrich and expresses predesigned, validated MISSION shRNA for GFP (SHC005), *CDK5* (TRCN0000021467), or *p35* (shp35-1: TRCN0000231936; shp35-2: TRCN0000231937; data that do not specify between shp35-1 or shp35-2 were obtained using the shp35-1 clone). pMD2.G envelope plasmid (Addgene, catalog no.12259), psPAX2 packaging plasmid (Addgene, catalog no.12260), pLVX lentiviral expression vector (Addgene, catalog no.133025), and pUltra vector containing tandem P2A/T2A self-cleaving peptide sequence (Addgene, catalog no. 24129) were obtained from Addgene. The P2A/T2A sequence and a downstream puromycin resistance gene were subcloned into the pLVX vector. The *CDK5* or *p35* coding sequence was subcloned into the pLVX vector upstream of the P2A/T2A sequence using appropriate primers and cDNA template obtained from 1° NK cells. The CDK5-K33T mutant transgene was subcloned into the pLVX vector using the *CDK5* vector as a template and using appropriate primers to convert the 33rd codon from *aaa* to *aca*, resulting in the K33T mutation that was validated previously (30).

### Lentiviral Transduction

HEK293T cells in a 10-cm plate were transfected with pMD2.G (1.25 μg), psPAX2 (3.75 μg), and either pLKO.1 or pLVX vectors (5 μg) using X-tremeGENE HP DNA transfection reagent (Roche). After 16 hours, media was replaced with fresh media. Viral supernatant was collected at 48 and 72 hours posttransfection and was filtered through a 0.45 μm PES syringe filter (Thermo Fisher Scientific), followed by concentration using Lenti-X Concentrator (Takara) following manufacturer's protocol. Lentivirus was resuspended in NK92 complete media. 1e6 NK92 cells were resuspended in 1 mL of lentivirus-containing media and polybrene (8 μg/mL) in a 12-well plate. Transduction was performed via “spinfection” (centrifugation at 1,000 × *g*, 90 minutes, 32°C). A total of 1 mL additional complete media was added to the well, and the plate was placed in the incubator overnight. Cells were then resuspended in fresh complete media and cultured for 48 hours, after which cells were resuspended in media containing puromycin (2 μg/mL) for positive selection of successfully transduced cells. Cells were resuspended in fresh media and puromycin every 2 days for a total duration of 6 days. Cells were then cultured in fresh media without puromycin.

### Flow Cytometry

Cells were washed with PBS and then stained with fluorophore-conjugated antibody for 15 minutes at room temperature or 40 minutes at 4°C. Cells were washed again with PBS and then analyzed using the CytoFLEX flow cytometer (Beckman Coulter).

### Western Blot Analysis

Cells were harvested and washed with PBS, then lysed with RIPA buffer (Millipore) containing Halt Protease/Phosphatase Inhibitor Cocktail and 5 mmol/L ethylenediaminetetraacetic acid (EDTA) (Thermo Fisher Scientific). Lysate was spun at 14,000 rpm for 15 minutes to pellet cell debris, and protein was quantified using the Pierce BCA Protein Assay Kit (Thermo Fisher Scientific). Protein lysates were diluted with RIPA buffer and 4x Laemmli buffer prepared with β-mercaptoethanol (Bio-Rad) to a final concentration of 3 μg protein/μL and heated to 95°C for 5 minutes in a heat block. Protein samples were run in 12% SDS-PAGE gels, followed by transfer onto 0.22 μm nitrocellulose membrane (Bio-Rad) using Towbin buffer with 20% methanol. Membranes were blocked using 5% milk diluted in Tris-buffered saline with 0.1% Tween 20 (TBST). Primary antibodies (Cell Signaling Technology) were generally diluted 1:1,000 in 5% BSA Fraction V (Thermo Fisher Scientific) in TBST as recommended by the manufacturer; GAPDH and β-actin antibody were purchased from Santa Cruz Biotechnology and diluted 1:10,000 in 5% BSA in TBST. Secondary antibodies (Cell Signaling Technology) were diluted in 5% milk in TBST. Membranes were imaged using either a Konica Minolta SRX-101A developer or a ChemiDoc XRS+ System imager (Bio-Rad).

### Kinase Activity Assay

CDK5 kinase activity was measured using the CDK5/p35 Kinase Enzyme System (Promega) and ADP-Glo Kinase Assay (Promega) as reported previously (23). Briefly, NK-cell lysate was prepared with RIPA buffer and diluted to a concentration of 1 mg/mL. A total of 200 μg protein lysate was precleared with protein A-magnetic beads (Cell Signaling Technology) and Rabbit (DA1E) IgG XP Isotype Control antibody (#3900, Cell Signaling Technology) at 4°C for 2 hours on a rotator, followed by rotating overnight incubation with 1:50 dilution of CDK5 antibody (#2506, Cell Signaling Technology). A total of 20 μL of protein A-magnetic beads were then added followed by rotation at 4°C for 3 hours. Immunoprecipitates were washed twice with lysis buffer and twice with kinase buffer (recipe as reported previously; ref. 23). Immunoprecipitates were resuspended in 30 μL of kinase buffer. 10 μL of protein solution were combined with DMSO and kinase buffer containing ATP and histone H1 substrate, for a final concentration of 5% DMSO, 10 μmol/L ATP, and 0.1 μg/μL histone H1. For roscovitine conditions, 5% DMSO was replaced with 50 μmol/L roscovitine. For recombinant CDK5/p35 conditions, CDK5 immunoprecipitates were replaced with 25 ng recombinant CDK5/p35. Samples were processed as described by the manufacturer's protocol. Magnetic beads were isolated using a magnetic and the solution was transferred to a 96-well luminescence plate. Luminescence was measured using a GloMax Discover Microplate Reader (Promega).

### Cell-cycle Analysis

NK92 cells were washed with PBS and then fixed with 70% cold ethanol, which was added dropwise to the cell pellet while vortexing. Cells were fixed at 4°C for 30 minutes, followed by two washes with PBS. Cells were resuspended in Propidium Iodide (PI)/RNase Staining Solution (Cell Signaling Technology) and analyzed using flow cytometry.

### Peripheral Blood Mononuclear Cell Isolation and NK-cell Expansion

Peripheral blood mononuclear cells (PBMC) were isolated from healthy donor blood (provided by the CWRU Hematopoietic Biorepository and Cellular Therapy Core) using Ficoll-Paque Plus (Cytiva) density gradient centrifugation. NK cells were expanded from PBMCs using K562 cells that express membrane-bound IL21 and 4-1BBL, as reported previously (31). Following 2 weeks of expansion, NK-cell purity was assessed using flow cytometry.

### Transcriptional Expression Analysis

Expanded NK cells were cultured in complete RPMI1640 media (10% FBS, 1% P/S) without IL2 for 48 hours. A total of 2e6 cells were harvested for RNA isolation using the RNeasy Plus Mini Kit (Qiagen). cDNA was obtained from RNA using iScript Reverse Transcription Supermix for qRT-PCR (Bio-Rad). PCR was conducted to amplify regions from the coding sequences of *CDK5*, *p35*, *p39*, and *ACTIN*. The specific forward and reverse primers used are as follows: CDK5-F: GATGTCGATGACCAGTTGAAGAG; CDK5-R: CGGACAGAAGTCGGAGAAGTA; p35-F: GCTCCTCCTCAGTCAAGAAAG; p35-R: TAGTGTGGGTCGGCATTTATC; p39-F: TGCCCGAGGAGAAGAAGAA; p39-R: CTTGAAAGACCTGCGTGAAGA; Actin-F: GGCACCACACCTTCTACAAT; Actin-R: CCTTAATGTCACGCACGATTTC. PCR products were run on a 2% agarose gel in TAE buffer. A total of 100 bp DNA ladder (New England Biolabs) was used as a reference. The gel was imaged using a Gel Doc XR+ System imager (Bio-Rad).

### qRT-PCR

RNA was isolated from NK92 cells and cDNA was generated as described for transcriptional expression analysis. qPCR was performed using iQ SYBR Green Supermix (Bio-Rad) and samples were run on the CFX96 Touch Real-Time PCR Detection System (Bio-Rad). The specific forward and reverse primers used are as follows: CDK5-F: GCTGGATGACGATGATG; CDK5-R: GCCTGACGATGTTCTTG; p35-F: CCTGTACCTCTCCTACTC; p35-R: GTGGGTCGGCATTTATC; GAPDH-F: CTGACTTCAACAGCGACACC; GAPDH-R: TAGCCAAATTCGTTGTCATACC.

### Proliferation and Viability Assays

A total of 250,000 shRNA-expressing NK92 cells were plated in 1 mL of complete media in separate wells of a 24-well plate for 48 hours, after which live cells were counted via trypan blue staining and a hemocytometer. Cells were also collected for analysis using APC Annexin V Apoptosis Detection Kit with PI (BioLegend), following manufacturer's protocol.

### Flow-based Cytotoxicity Assay

Jeko-1 cells were labeled with the fluorescent Cell Proliferation Dye eFluor 670 (Thermo Fisher Scientific) following manufacturer's protocol. NK92 cells were resuspended in fresh RPMI media and mixed with 30,000 Jeko-1 cells per well at different effector:target (E:T) ratios in a flat-bottom 96-well plate. Wells with Jeko-1 cells only were used to measure spontaneous cell death. After 16 hours of incubation, cells were transferred to a V-bottom plate and washed with PBS. Cells were stained with 80 μL of PI (Cell Signaling Technology) at room temperature in the dark for 20 minutes. A total of 50 μL of PBS were subsequently added, followed by data collection using flow cytometry. 10,000 eFluor670+ events corresponding to labeled Jeko-1 cells were recorded per sample. PI+ percentage of labeled Jeko-1 cells corresponding to cancer cell death was graphed. For cytotoxicity experiments with TGFβ, NK92 cells were cultured in 2.5 ng/mL TGFβ for 48 hours. NK92 cells were resuspended in RPMI media and mixed with Jeko-1 cells as described above but in the presence of either PBS or 2.5 ng/mL TGFβ for 16 hours of incubation.

### Calcein AM–based Cytotoxicity Assay

Murine NK cells of similar genotype isolated from splenocytes of individual mice were pooled and cultured in RPMI media supplemented with 10% FBS, 1% P/S, 1 mmol/L sodium pyruvate (Gibco), 1× MEM non-essential amino acids (Gibco), 55 μmol/L β-mercaptoethanol (Gibco), 25 mmol/L HEPES, and 1,000 U/mL recombinant human IL2 (BioLegend), based on previously published protocol for *ex vivo* culture of mNK cells (32). B16F10 cells were labeled with Calcein AM cell-permeant dye (Thermo Fisher Scientific) following manufacturer's protocol. Murine NK cells were resuspended in fresh DMEM and mixed with 30,000 B16F10 cells per well at different effector:target ratios in a U-bottom 96-well plate. Wells with B16F10 cells only were used to measure spontaneous calcein release. Wells with B16F10 cells and 0.1% Triton X-100 were used to measure maximum calcein release. After 4 hours of incubation, cells were spun at 400 × *g* for 5 minutes. A total of 100 μL supernatant was transferred to a black-walled, clear-bottom 96-well plate. Fluorescence intensity of supernatant containing released calcein was measured using a GloMax Discover Microplate Reader (Promega). Cytotoxicity was calculated using the following formula: [(experimental release value − spontaneous release value)/(max release value − spontaneous release value)] × 100.

### Cytokine Release Assays

Supernatant from human or murine NK-cell culture was collected and stored at −80°C. The flow cytometry–based LEGENDplex Human CD8/NK Panel and Mouse Inflammation Panel (BioLegend) cytokine release assays were used to measure the concentration of different cytokines and lytic enzymes, following manufacturer's protocol.

### Statistical Analysis

All statistical analyses were performed using GraphPad Prism 9.4. The statistical methods used are indicated in figure legends. In all figures, data are presented as mean ± SD, unless stated otherwise. *n* = 3 technical or biological replicates were used in all experiments except as noted in figure legend. For *ex vivo* murine NK cytotoxicity experiments, NK cells from several mice of the same genotype were pooled to have sufficient cells to perform the assay. For murine NK-cell maturation marker or activating/inhibitory receptor expression analysis, flow cytometry plots/histograms are representative of samples obtained from multiple mice. Western blots are representative of multiple experiments. All experiments were conducted at least twice to ensure reproducibility.

### Data Availability Statement

The data generated in this study are available within the article. Further inquiries may be directed to the corresponding author.

## Results

### CDK5 and p35 are Expressed in NK Cells

First, we determined whether CDK5 and its primary known coactivators, p35 and p39, are expressed in NK cells (Fig. 1A). Using qRT-PCR with RNA isolate obtained from primary (1°) human NK cells, we observed that *CDK5* and *p35*, but not *p39*, are transcriptionally expressed in these cells (Fig. 1B). Using protein lysates, we then confirmed that CDK5 and p35, but not p39, are translationally expressed in 1° NK cells (Fig. 1C). Mouse brain lysate (MBL) from WT mice and DAOY human medulloblastoma cell line served as positive controls, while MBL from p35 k/o mice served as a negative control. Next, we wanted to confirm that the CDK5 in NK cells is kinase-active. We immunoprecipitated CDK5 from NK92 cells, a human NK-cell line, then mixed it with histone H1, a known CDK5 substrate. Using the ADP-Glo kinase assay, we were able to detect significant levels of CDK5/p35 kinase activity in NK92 cells (Fig. 1D). Next, we assessed whether NK-cell activation affects CDK5 and p35 expression levels. 1° NK cells were cultured for 48 hours without the presence of activating cytokines, followed by the addition of 200 U/mL IL2. While ERK1/2 phosphorylation occurs rapidly within the first 2 hours of IL2 activation, p35 expression is low and appears to decrease reduced at this time. p35 expression levels then increase starting from 4 hours up to at least 24 hours after the introduction of IL2 (Fig. 1E). p35 expression increases most significantly as ERK1/2 phosphorylation is already diminishing, suggesting that IL2 stimulation results in decreased p35 expression, followed by a late-stage induction of p35. The levels of p25 appear generally increased at all timepoints after the addition of IL2, compared with 0 hour, while there are no significant changes in the pattern of CDK5 expression. Taken together, these results show that CDK5 and p35 are expressed in NK cells, that CDK5 is kinase-active, and that p35 may be involved in NK-cell activation.

### CDK5 Knockdown Inhibits NK-cell Survival and p35 Knockdown Increases Cytotoxicity

**FIGURE 2:**
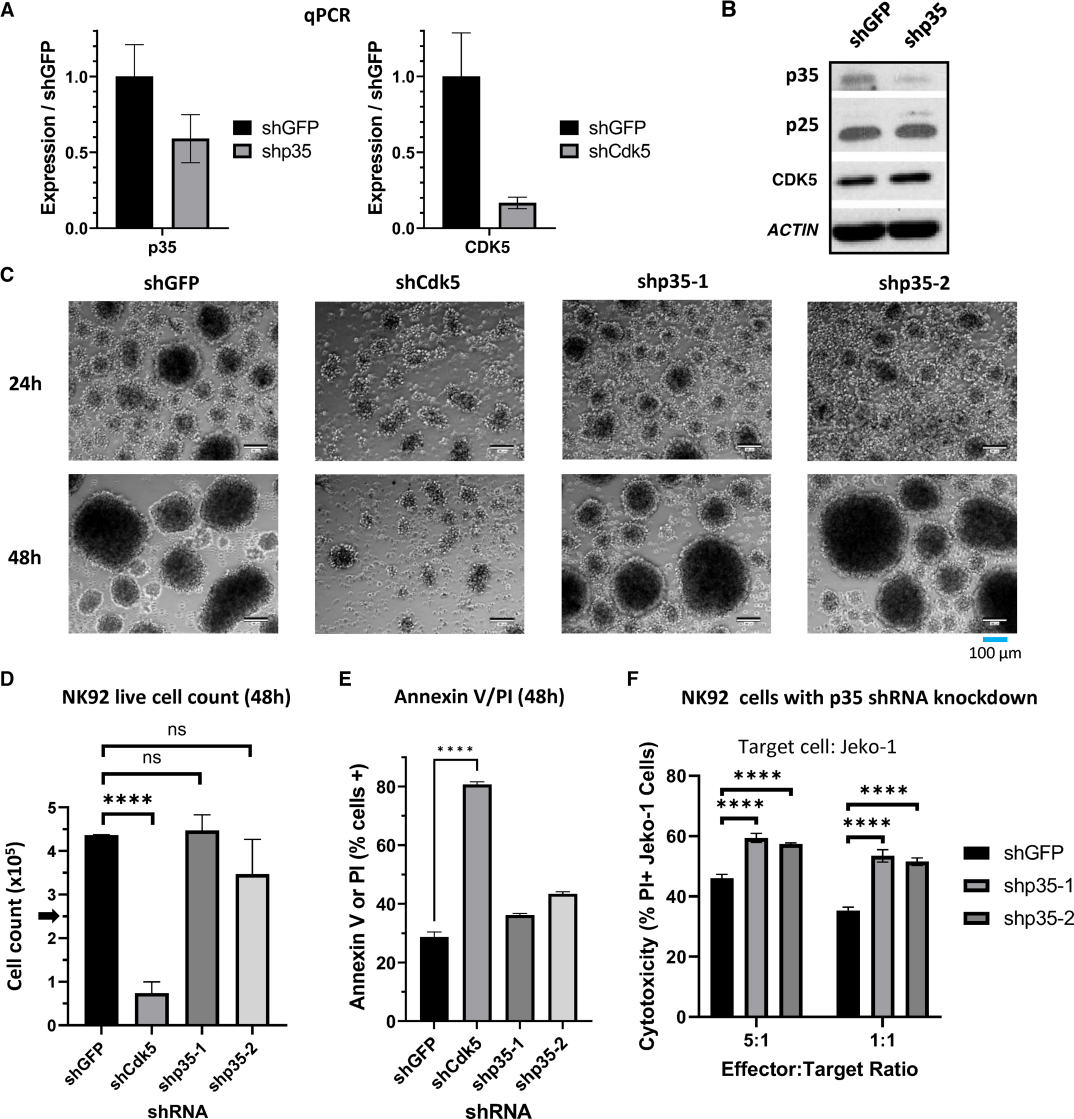
CDK5 knockdown inhibits NK92 survival while p35 knockdown increases NK92 cytotoxicity. **A,** qRT-PCR results using RNA isolated from NK92 cells transduced with *p35* shRNA (shp35) or *CDK5* shRNA (shCdk5) compared with shGFP control. *GAPDH* was used as the housekeeping gene. Graphs display mean ± SD, *n* = 3 technical replicates. **B,** Western blot analysis of protein from NK92 cells transduced with *p35* shRNA (shp35) or negative control *GFP* shRNA (shGFP). **C,** Phase contrast microscopy of NK92 cells transduced with *CDK5* shRNA or one of two *p35* shRNA clones, compared with shGFP control. Images are 10x magnification; scale bar, 100 μm. **D,** NK92 cells were plated in complete media with a starting count of 250,000 cells. Live cell count was obtained after 48 hours cell culture via trypan blue staining and manual counting on a hemocytometer. ****, *P* < 0.0001. Graphs display mean ± SD, *n* = 3 biological cultures, one-way ANOVA with Dunnett multiple comparisons test. **E,** NK92 cells were stained with Annexin V and PI and analyzed via flow cytometry. Percentage of cells staining positive for Annexin V and/or PI were graphed. ****, *P* < 0.0001. Graphs display mean ± SD, *n* = 3 biological cultures, one-way ANOVA with Dunnett multiple comparisons test. **F,** NK92 cells were cocultured with fluorescently-labeled Jeko-1 cells for 16 hours to measure the effect of p35 knockdown on cytotoxicity. Cells were stained with PI and gated on the labeled cancer cells to measure cancer cell death. ****, *P* < 0.0001. Graphs display mean ± SD, *n* = 3 biological cocultures, two-way ANOVA with Dunnett multiple comparisons test.

To determine how CDK5/p35 affect NK-cell function, we transduced NK92 cells with shRNA vectors to knock down either *CDK5* (shCdk5), *p35* (clones shp35-1 or shp35-2), or *GFP* as a negative control (shGFP). *CDK5* knockdown was confirmed via qPCR (Fig. 2A), while *p35* knockdown was confirmed via qPCR and Western blot analysis (Fig. 2A and B). p35 knockdown does not appear to affect CDK5 or p25 expression compared with shGFP control cells (Fig. 2B). However, CDK5 knockdown cells displayed impaired proliferation and survival. Phase contrast microscopy showed that while shGFP and shp35 cells clustered and expanded healthy, shCdk5 cells did not cluster and appeared visibly unhealthy (Fig. 2C). shCdk5 viable cell count decreased after 48 hours while shp35 cells continued proliferating similar to shGFP cells (Fig. 2D). Annexin V and PI staining confirmed significantly lower viability in shCdk5 cells compared with shGFP and shp35 cells (Fig. 2E). Therefore, we continued our analysis of CDK5/p35 function using the shp35 NK92 cells. The primary role of NK cells is cell-mediated cytotoxicity against aberrant target cells. Therefore, we used a flow cytometry–based cytotoxicity assay to assess the effect of p35 knockdown on NK92-cell cytotoxicity against the Jeko-1 mantle cell lymphoma cells. Notably, p35 knockdown using either shRNA clone appeared to significantly increase NK-cell cytotoxicity against the cancer cells by up to 20% compared with the shGFP control (Fig. 2F). These results suggest that while CDK5 expression appears to be necessary for NK92 cell viability, its kinase activity through p35 does not affect viability, but rather, CDK5/p35 activity has a separate role in NK92-cell cytotoxic function.

### NK Cells from p35 k/o Mice Show Increased Cytotoxicity Against Murine Cancer Cells

**FIGURE 3:**
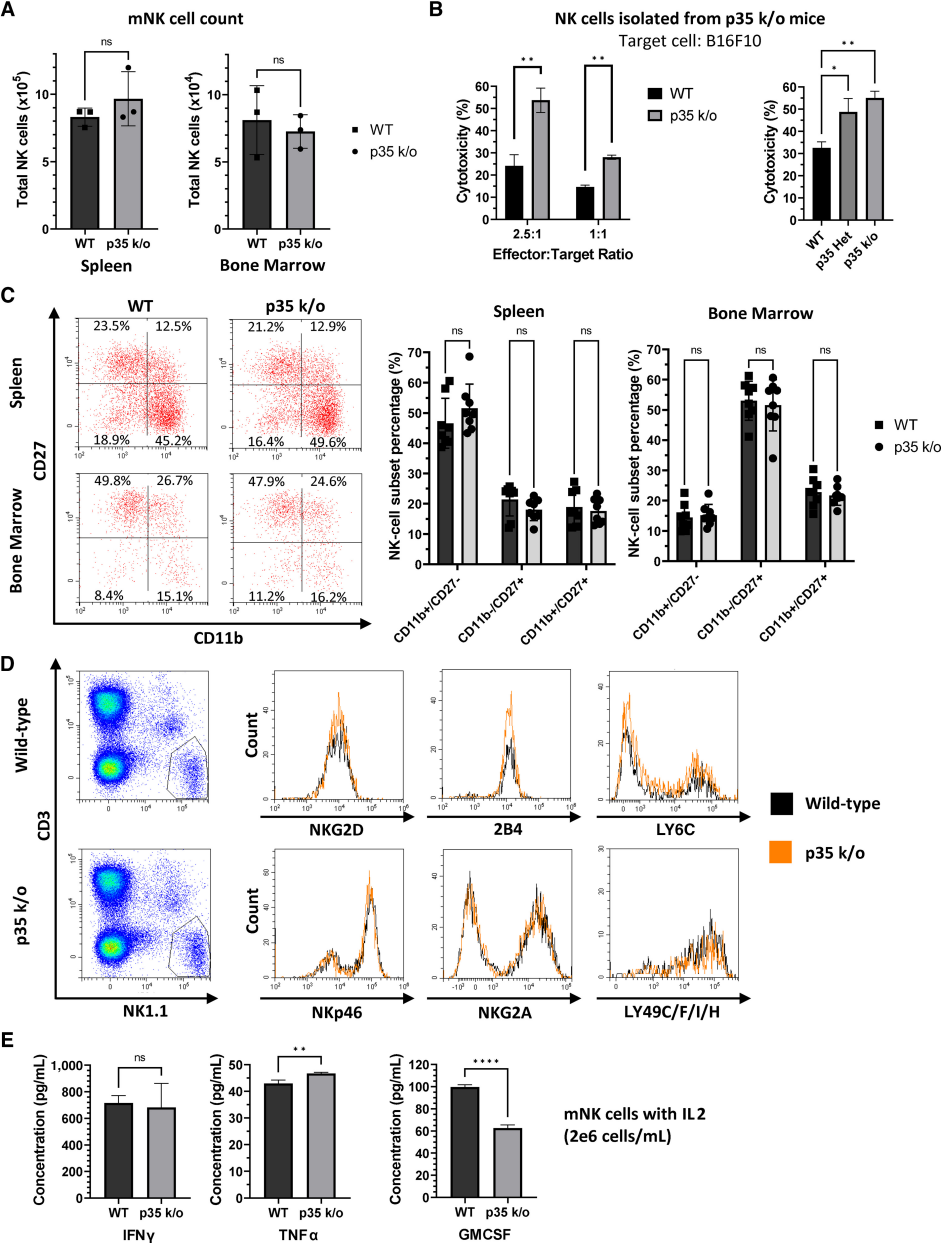
Murine NK cells from p35 k/o mice display heightened cytotoxicity. **A,** Murine splenocytes or bone marrow cells from WT or p35 k/o mice were stained for CD3 and NK1.1 and mixed with counting beads, then analyzed via flow cytometry. Gating on NK1.1^+^CD3^−^ cells, as well as the counting beads, allowed determination of mNK concentration and the total mNK cell count in each compartment. ns, not significant. Graphs display mean ± SD, *n* = 3 mice, unpaired two-tailed *t* test. **B,** mNK cells were isolated from WT, p35 k/o, or p35 Het mice and cocultured with calcein AM-labeled B16F10 cells at different E:T ratios for 4 hours. Fluorescence intensity of supernatant corresponding to calcein release from dead cells was measured to calculate NK-mediated cytotoxicity. **, *P* < 0.01. Graphs display mean ± SD, *n* = 3 biological cocultures (*n* = 2 for WT 1:1 ratio, p35 k/o 1:1 ratio, and p35 k/o in WT versus p35 Het versus p35 k/o experiment), and multiple unpaired two-tailed *t* tests with correction for multiple comparisons using Holm-Šídák method (WT vs. p35 k/o) or one-way ANOVA with Tukey multiple comparisons test (WT vs. p35 Het vs. p35 k/o). **C,** Murine splenocytes or bone marrow cells were stained for CD11b, CD27, CD3, and NK1.1 and analyzed via flow cytometry after gating on NK1.1^+^CD3^−^ cells. Representative flow plots are shown. Percentages of mNK cells in each subset (CD11b^+^CD27^−^, CD11b^−^CD27^+^, and CD11b^+^CD27^+^) for each mouse were recorded and graphed. ns, not significant. Graphs display mean ± SD, *n* = 8 mice, multiple unpaired two-tailed *t* tests with correction for multiple comparisons using Holm-Šídák method. **D,** Murine splenocytes were stained for CD3, NK1.1, and NKG2D, NKp46, NKG2A, 2B4, LY6C, or LY49C/F/I/H. Representative histograms of activating/inhibitory receptor expression are shown, gated on live NK1.1^+^CD3^−^ cells. **E,** Isolated mNK cells pooled from multiple mice were seeded at a concentration of 2e6 cells/mL and cultured *ex vivo* in RPMI media supplemented with 1,000 U/mL IL2. An additional 1,000 U/mL IL2 was added 2 days later without changing the media, followed by collection of supernatant after culture for an additional 2 days. Cytokine concentration was determined using a flow-based, multiplex murine cytokine release assay. ns, not significant; *, *P* < 0.05; **, *P* < 0.01. Graphs display mean ± SD, *n* = 3 biological cultures, unpaired two-tailed *t* tests.

Given the significant effect of p35 knockdown on human NK-cell cytotoxicity, we wanted to evaluate the phenotype of mNK cells from p35 k/o mice. First, we measured the total mNK cell count in the spleen and bone marrow compartments of WT versus p35 k/o mice. There was no apparent difference in the average number of NK cells in spleen or bone marrow in p35 k/o mice (Fig. 3A). Next, we wanted to evaluate the cytotoxic function of p35 k/o NK cells. After isolating mNK cells from bulk splenocytes of WT and p35 k/o mice and activating them *ex vivo* with IL2, we cocultured them with the B16F10 murine melanoma cell line at different E:T ratios and observed a significant increase in NK cell–mediated cytotoxicity by p35 k/o mNK cells relative to the WT control (Fig. 3B). We also compared the cytotoxicity of mNK cells from p35 ± (p35 Het) mice to that of WT and p35 k/o mNK cells. p35 Het mNK cells appeared to exhibit intermediate cytotoxicity that was higher than that of WT mNK cells but lower than that of p35 k/o mNK cells (Fig. 3B).

Expression of the CD11b and CD27 maturation markers are used to distinguish between mNK cell subtypes. CD11b^low^/CD27^+^ and CD11b^hi^/CD27^+^ mNK cells have higher cytotoxic function than CD11b^hi^/CD27^−^ mNK cells (33). Therefore, we wanted to determine whether the higher cytotoxicity of p35 k/o mNK cells could be attributed to a greater percentage of the CD11b^+^/CD27^−^ subtype among mNK cells. Using splenocytes and bone marrow cells obtained from WT and p35 k/o mice, we did not observe any significant changes in the percentage of mNK cell subtypes (Fig. 3C). We were also interested in analyzing whether the surface expression of various mNK activating or inhibitory receptors differed between WT and p35 k/o mice. However, using flow cytometry, we did not notice any significant differences in the expression of various well-characterized activating/inhibitory receptors on the mNK cells, including NKp46, NKG2D, NKG2A, 2B4, LY6C, LY49C/F/I/H (Fig. 3D). We also evaluated any differences in cytokine secretion between WT and p35 k/o mNK cells. After isolation of mNK cells, we cultured them *ex vivo* with 1,000 U/mL IL2 for 4 days, after which supernatant was collected for measurement of cytokine release. p35 k/o mNK cells appear to secrete marginally higher levels of TNFα, lower levels of GMCSF, and similar levels of IFNγ compared with WT mNK cells (Fig. 3E).

### Overexpression of a Kinase-dead CDK5 Mutant Increases NK Cytotoxicity

**FIGURE 4:**
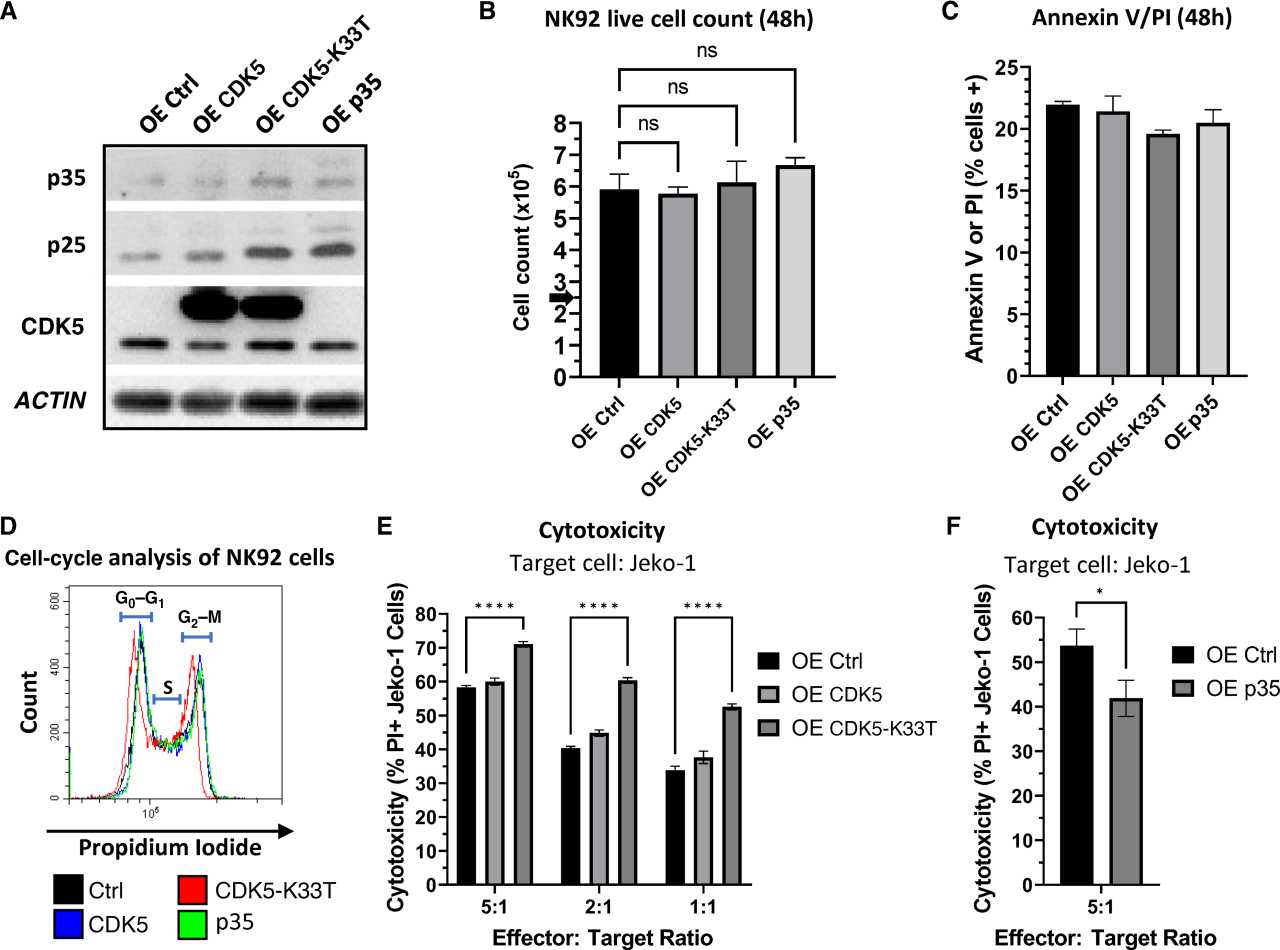
Overexpression of kinase-dead CDK5 increases NK92 cytotoxicity while overexpression of p35 decreases cytotoxicity. **A,** Western blot analysis of protein from NK92 cells overexpressing CDK5 (OE CDK5), kinase-dead mutant CDK5 (OE CDK5-K33T), p35 (OE p35), or empty vector control (OE Ctrl). **B,** NK92 cells were plated in complete media with a starting count of 250,000 cells. Live cell count was obtained after 48 hours cell culture via trypan blue staining and manual counting on a hemocytometer. ns, not significant. Graphs display mean ± SD, *n* = 3 biological cultures, one-way ANOVA with Dunnett multiple comparisons test. **C,** NK92 cells were stained with Annexin V and PI and analyzed via flow cytometry. Percentage of cells staining positive for Annexin V and/or PI were graphed. Graphs display mean ± SD, *n* = 3 biological cultures. **D,** NK92 cells were fixed in 70% ethanol and stained with PI, then analyzed via flow cytometry. G_0_–G_1_, S, and G_2_–M phases of the cell cycle are indicated. **E,** NK92 cells (OE Ctrl, OE CDK5, OE CDK5-K33T) were cocultured with fluorescently-labeled Jeko-1 cells for 16 hours at different E:T ratios, followed by staining with PI and gating on labeled cancer cells to measure cancer cell death via flow cytometry. ****, *P* < 0.0001. Graph displays mean ± SD, *n* = 3 biological cocultures, two-way ANOVA with Dunnett multiple comparisons test. **F,** NK92 cells (OE Ctrl, OE p35) were cocultured with fluorescently-labeled Jeko-1 cells for 16 hours at 5:1 E:T ratio, followed by staining with PI and gating on labeled cancer cells to measure cancer cell death via flow cytometry. *, *P* < 0.05. Graph displays mean ± SD, *n* = 3 biological cocultures, unpaired two-tailed *t* test.

Because p35 knockdown/knockout resulted in increased NK-cell cytotoxicity, we wanted to examine the phenotypic effect of p35 or CDK5 overexpression in these cells. We transduced NK92 cells to exogenously express either CDK5, p35, or a K33T kinase-dead mutant of CDK5 that cannot bind ATP but can still bind p35 (CDK5-K33T; ref. 30). We confirmed protein overexpression via Western blot analysis (Fig. 4A). p25 appears to increase with p35 overexpression, and in addition, we observed an increase in both p35 and p25 with expression of the CDK5-K33T mutant. Expression of this dominant-negative CDK5 mutant has been previously shown to increase the stability of p35 by 2- to 3-fold, which may explain our observation here (9). As CDK5 knockdown inhibited NK92 cell survival, we wanted to assess whether overexpression of CDK5, p35, or the CDK5-K33T mutant would affect viability, proliferation, or the cell cycle. After seeding the cells at the same starting number and concentration, we did not observe any significant difference in proliferation after 48 hours, and cell viability between the NK92 populations was similar (Fig. 4B and C). We fixed the NK92 cells using ethanol and stained them with PI, then analyzed the cells via flow cytometry. We did not observe any notable changes in the proportion of NK92 cells in the G_0_–G_1_, S, or G_2_–M phases of the cell cycle (Fig. 4D). Next, we assessed the cytotoxic function of these NK92 cells using Jeko-1 cytotoxicity assays. Intriguingly, while overexpression of WT CDK5 does not appear to affect cytotoxicity significantly, we observed a significant increase in cytotoxicity mediated by NK92 cells overexpressing the CDK5-K33T mutant compared with control (Fig. 4E). Meanwhile, p35-overexpressing cells displayed a notable decrease in cytotoxicity at the 5:1 E:T ratio (Fig. 4F). This negative effect of p35 overexpression on cytotoxic function correlates with the previously observed increase in cytotoxicity upon knockdown/knockout of p35. The increase in cytotoxicity from expression of kinase-dead CDK5 further supports the hypothesis that p35-mediated CDK5 activity regulates NK-cell cytotoxicity.

### TGFβ Induces p35 Expression Earlier than IL2 While Impairing NK Cytotoxicity

**FIGURE 5:**
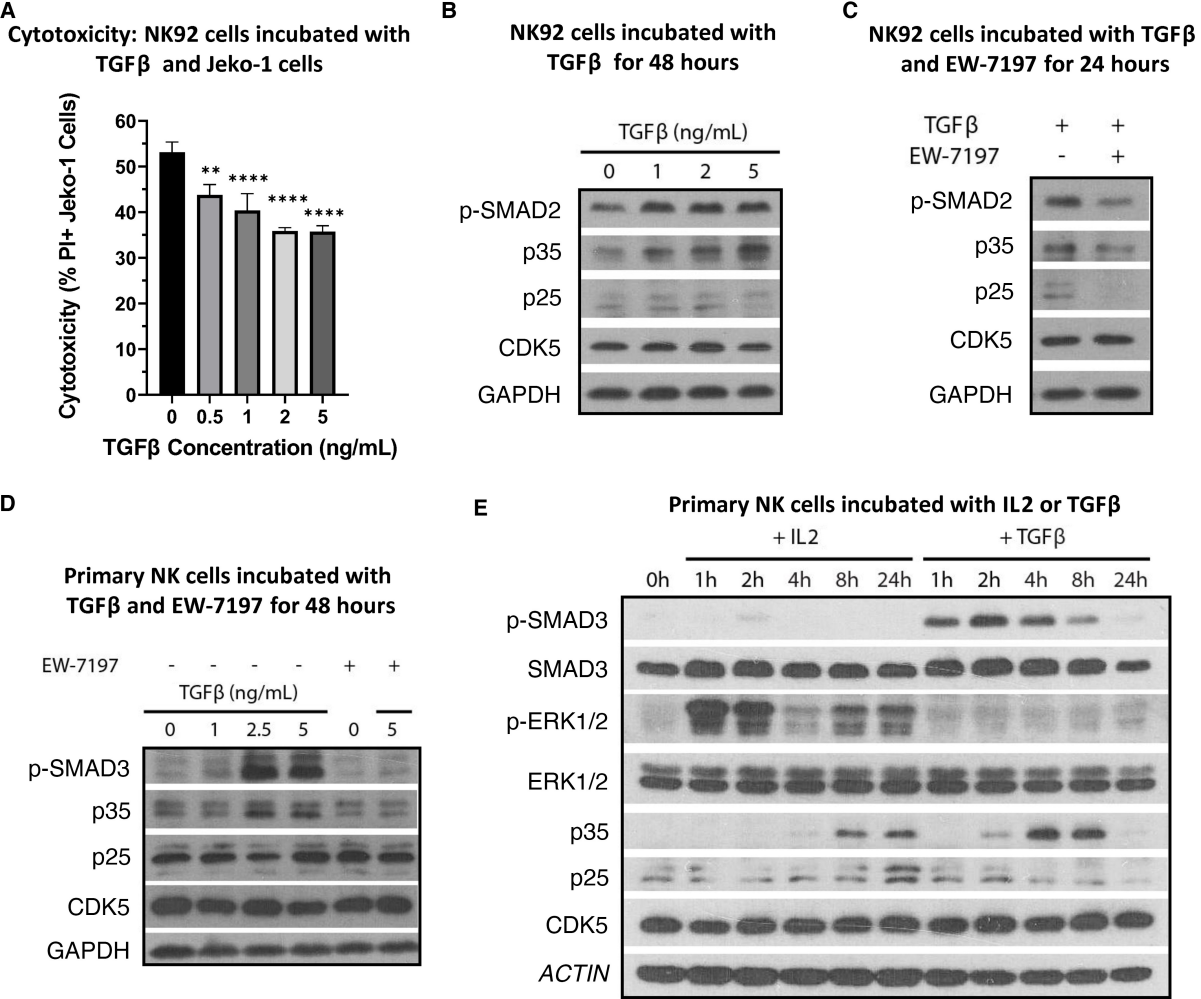
TGFβ induces p35 expression in a dose- and time-dependent manner. **A,** NK92 cells were incubated with fluorescently-labeled Jeko-1 cells and different concentrations of TGFβ for 16 hours. Cells were stained with PI and gated on labeled cancer cells via flow cytometry to measure cancer cell death. **, *P* < 0.01; ****, *P* < 0.0001. Graphs display mean ± SD, *n* = 3 biological cocultures, one-way ANOVA with Dunnett multiple comparisons test. **B,** NK92 cells were incubated with different concentrations of TGFβ in complete media for 48 hours. Protein was collected and analyzed via Western blot analysis. **C,** NK92 cells were incubated with 5 ng/mL TGFβ in complete media for 24 hours with or without the TGFβ receptor inhibitor EW-7197 (5 ng/mL). Protein was collected and analyzed via Western blot analysis. **D,** 1° NK cells were incubated in complete media with different concentrations of TGFβ and/or 5 ng/mL EW-7197 for 48 hours. Protein was collected and analyzed via Western blot analysis. **E,** 1° NK cells were cultured in cytokine-free media for 48 hours, followed by culture with either 200 U/mL IL2 or 5 ng/mL TGFβ. Protein lysate was collected at the indicated timepoints.

TGFβ has been reported to induce CDK5 and p35 expression in breast cancer cells (32). It is also well known that TGFβ impairs NK-cell cytotoxicity (5, 6). We analyzed whether TGFβ-mediated inhibition of NK-cell cytotoxicity could be linked to CDK5/p35 activity. As expected, coculturing NK92 cells with Jeko-1 cells in the presence of TGFβ results in a dose-dependent decrease in cytotoxicity (Fig. 5A). At the same time, culturing NK92 cells with TGFβ for 48 hours demonstrates a dose-dependent increase in p35 expression (Fig. 5B). Neither expression of p25 nor CDK5 changed in any discernible pattern. Next, using a known TGFβ receptor inhibitor, EW-7197, we cultured NK92 cells with TGFβ and with and without EW-7197 (34). The presence of EW-7197 inhibited TGFβ signaling, as seen by the decrease in p-SMAD2, and it inhibited p35 expression as well (Fig. 5C). Next, we confirmed the same effects of TGFβ and EW-7197 in 1° NK cells using additional concentrations of TGFβ. As observed in NK92 cells, p35 expression was induced by TGFβ in a dose-dependent manner, and the addition of EW-7197 reversed this effect (Fig. 5D). We previously observed that IL2, an activating cytokine, resulted in a late-stage induction of p35 expression. As TGFβ and IL2 have generally opposite effects on NK-cell activation and cytotoxicity, we wanted to compare the length of time required for p35 induction in response to TGFβ versus IL2. 1° NK cells were cultured without cytokine for 48 hours, after which either IL2 (200 U/mL) or TGFβ (5 ng/mL) was added. We observed a remarkable difference in the time to induction for p35 in response to these cytokines. While p35 does not obviously begin to increase until 4 hours after addition of IL2, similar to what we previously observed, p35 begins to increase only 2 hours after addition of TGFβ (Fig. 5E). This is despite p-ERK1/2 reaching its highest level only 1 hour after addition of IL2, while p-SMAD3 reaches its highest levels at 2 hours after addition of TGFβ. Furthermore, peak expression of p35 is reached at 4–8 hours after addition of TGFβ, whereas its peak expression is much later for IL2, up to 24 hours after addition of IL2. These results strongly suggest that p35 expression is regulated downstream of TGFβ signaling.

### TGFβ Inhibition of Cytotoxicity is Partially Reversed by p35 Knockdown or CDK5-K33T Overexpression

**FIGURE 6:**
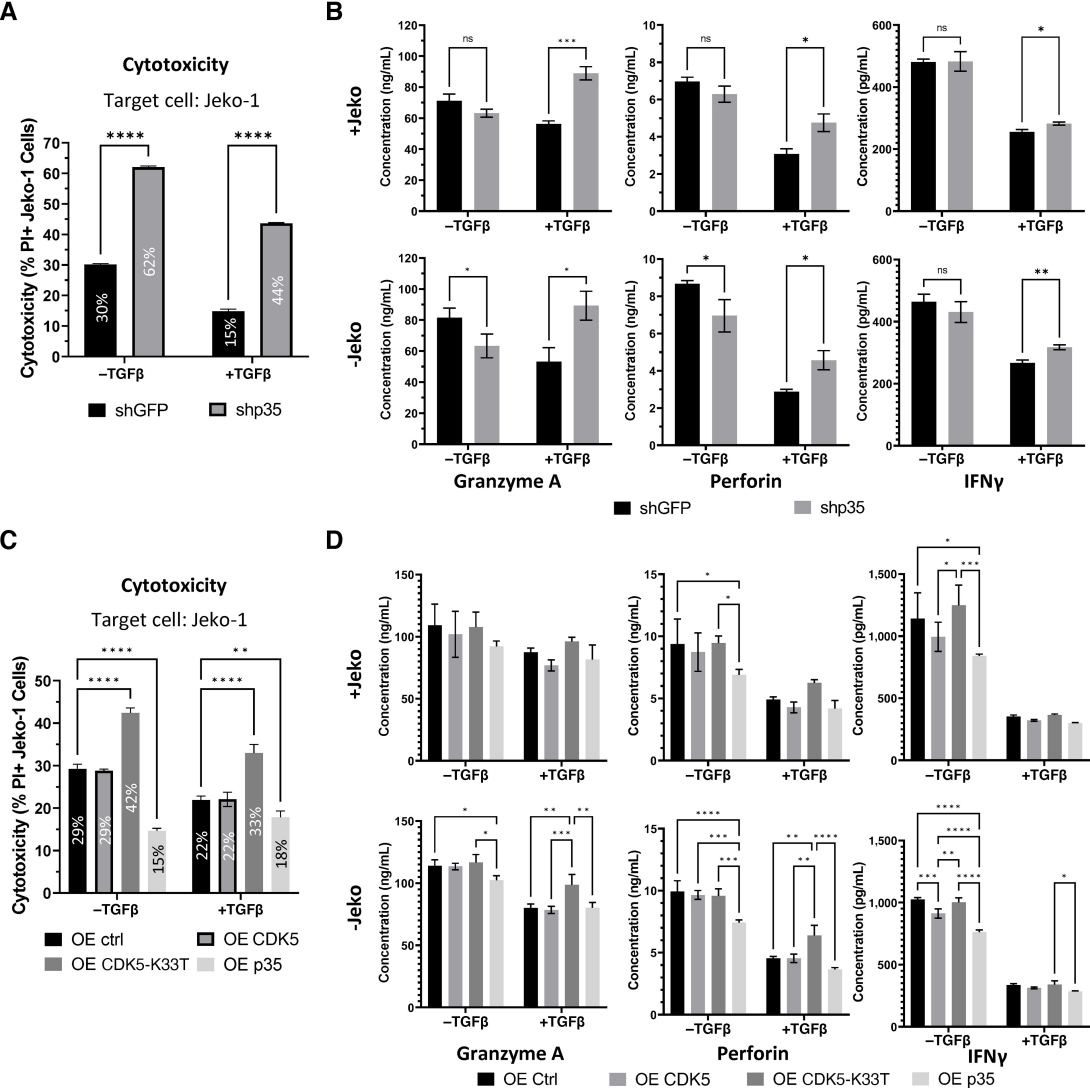
NK92 cells with p35 knockdown or CDK5-K33T overexpression mitigate TGFβ-induced inhibition of cytotoxicity. **A,** NK92 cells (shGFP or shp35) were cultured for 48 hours in the presence of 2.5 ng/mL TGFβ or PBS (−TGFβ). They were then cocultured with fluorescently-labeled Jeko-1 cells at 5:1 E:T ratio for 16 hours in the continued presence of 2.5 ng/mL TGFβ or PBS (−TGFβ). Cells were stained with PI and gated on labeled cells to measure cancer cell death. Average cytotoxicity percentage is overlaid on the corresponding bar. ****, *P* < 0.0001. Graphs display mean ± SD, *n* = 3 biological cocultures, multiple unpaired two-tailed *t* tests with correction for multiple comparisons using Holm-Šídák method. **B,** Supernatant from NK92 cell culture (shGFP or shp35) with or without Jeko cells was collected after 16 hours. Cytokine concentration was determined using a flow-based, multiplex human cytokine release assay. ns, not significant; *, *P* < 0.05; **, *P* < 0.01; ***, *P* < 0.001. Graphs display mean ± SD, *n* = 3 biological cocultures, multiple unpaired two-tailed *t* tests with correction for multiple comparisons using Holm-Šídák method. **C,** NK92 cells (OE ctrl, OE CDK5, OE CDK5-K33T, or OE p35) were cultured for 48 hours in the presence of 2.5 ng/mL TGFβ or PBS (−TGFβ). They were then cocultured with fluorescently-labeled Jeko-1 cells at 5:1 E:T ratio for 16 hours in the continued presence of 2.5 ng/mL TGFβ or PBS (−TGFβ). Cells were stained with PI and gated on labeled cells to measure cancer cell death. Average cytotoxicity percentage is overlaid on the corresponding bar. **, *P* < 0.01; ****, *P* < 0.0001. Graphs display mean ± SD, *n* = 3 biological cocultures, two-way ANOVA with Tukey multiple comparisons test. **D,** Supernatant from NK92 cell culture (OE ctrl, OE CDK5, OE CDK5-K33T, or OE p35) with or without Jeko cells was collected after 16 hours. Cytokine concentration was determined using a flow-based, multiplex human cytokine release assay. *, *P* < 0.05; **, *P* < 0.01; ***, *P* < 0.001; ****, *P* < 0.0001. Graphs display mean ± SD, *n* = 3 biological cocultures, two-way ANOVA with Tukey multiple comparisons test.

As TGFβ signaling appears to quickly induce the expression of p35 and is known to impair NK cytotoxic function, we wanted to assess how p35 knockdown, p35 overexpression, or CDK5-K33T overexpression would affect NK92 cytotoxicity when these cells are cultured in the presence of TGFβ. While the cytotoxicity of both shGFP and shp35 NK92 cells decreased with TGFβ treatment, shp35 NK92 cell cytotoxicity decreased by less than one-third relative to the −TGFβ condition (62% to 44%) while the cytotoxicity of shGFP NK92 cells decreased by approximately one-half (30% to 15%; Fig. 6A). Thus, shp35 NK92 cells still retained significantly higher cytotoxicity than control after treatment with TGFβ. NK92 cell cytokine release was measured using supernatant collected from these cytotoxicity cocultures, as well as supernatant from NK92 cells that were plated without cancer cells. While shp35 secretion of GRANZYME A (GrA), PERFORIN, and IFNγ does not appear much different from shGFP under −TGFβ conditions, shp35 cells secrete significantly higher levels of these proteins than shGFP cells under +TGFβ conditions (Fig. 6B). TGFβ causes decreased secretion of PERFORIN and IFNγ in both shp35 and shGFP cells, but the control cells appear to be impacted by this much more than the shp35 cells. In addition, TGFβ does not seem to impair shp35 secretion of GrA compared with that of control. These observations could be seen in both +Jeko and −Jeko cultures, suggesting that the effects of p35 knockdown and TGFβ are independent of cell-to-cell contact with a target cell.

Meanwhile, whereas the cytotoxicity of CDK5-K33T NK92 cells decreased from 42% to 33% when TGFβ was added, Ctrl and CDK5-overexpressing NK92 cells decreased in cytotoxicity from 29% to 22% (Fig. 6C). Thus, NK92 cells that express dominant-negative CDK5 retain higher cytotoxicity than control or WT CDK5 under TGFβ immunosuppression. Interestingly, the p35-overexpressing NK92 cells do not display a corresponding decrease in cytotoxicity when cultured with TGFβ, suggesting that TGFβ treatment does not further contribute to the effect of p35 overexpression. Differences in cytokine and lytic enzyme secretion from these NK92 cells could be discerned. p35 overexpression resulted in significantly decreased secretion of GrA, PERFORIN, and IFNγ in −TGFβ conditions, although for GrA in the +Jeko culture, this was not statistically significant (Fig. 6D). For the CDK5-K33T cells, increased GrA and PERFORIN secretion relative to control were readily observed in the +TGFβ condition but not as much in the −TGFβ condition, similar to what we previously observed with shp35 cells relative to shGFP. These increases are not statistically significant in the +Jeko cultures.

Taken together, these results suggest that TGFβ inhibition of NK-cell cytotoxicity is mediated at least in part through p35 and that downregulation of p35 partially reversed TGFβ-mediated inhibition of cytotoxicity in NK cells.

## Discussion

Though CDK5 is expressed ubiquitously, expression of p35 or p39, the primary coactivators of CDK5 kinase activity, are mostly reported in neuronal cells (8). However, further investigations have identified expression of p35 and a plethora of divergent roles for CDK5/p35 in many non-neuronal cell types, including cancer cells and immune cells (18–20, 35). In neutrophils, CDK5/p35 activity appears to contribute to the GTP-induced secretory response, which includes the secretion of the antimicrobial protein lactoferrin (18). In macrophages, CDK5/p35 suppresses the production of IL10 (19). In T cells, CDK5/p35 activity has been tied to multiple roles, including activation downstream of T-cell receptor stimulation and T cell–mediated neuroinflammation, the control of gene expression of IL2 and regulation of regulatory T cell development (20, 21, 36). Yet, a role for CDK5/p35 in NK cells has not been previously reported, nor has CDK5/p35 been linked to the negative regulation of immune cell–mediated cytotoxicity against cancer cells. In this study, we establish for the first time that inhibition of p35 leads to increased NK cell–mediated cytotoxicity and also points to a possibility that p35 is involved in TGFβ-mediated NK-cell dysfunction.

We observed that p35 knockdown in NK92 cells and p35 k/o in mNK cells resulted in significantly higher cytotoxicity against cancer cell lines, although this decreased p35 expression did not affect NK92 cell proliferation and viability or mNK cell count, subset distribution, or expression of various activating/inhibitory receptors. p35 k/o mNK cells stimulated with IL2 secreted similar levels of IFNγ and moderately higher levels of TNFα and significantly lower levels of GMCSF *ex vivo*. GMCSF was shown to have no significant effect on NK cell–mediated cytotoxicity, while IFNγ and TNFα are known to synergistically increase NK-cell cytotoxicity (37, 38).

Meanwhile, expression of the CDK5-K33T kinase-dead mutant markedly increased NK-cell cytotoxicity, while overexpression of WT CDK5 did not appear to have any significant effects on cytotoxicity. This may be explained by the fact that, while the dominant-negative CDK5-K33T cannot bind ATP, it is still capable of binding p35 (30). In the original study in which CDK5-K33T was first utilized to study CDK5/p35 function in neuronal cells, CDK5-K33T expression greatly abolished p35-associated kinase activity (30). We believe the same effect is happening when we express CDK5-K33T in NK92 cells. The highly expressed CDK5-K33T competes with endogenous CDK5 in binding to p35, vastly reducing the amount of p35 available in the cell to bind to kinase-capable, endogenous CDK5. The expected effect would be similar to that of p35 knockdown, and indeed, we observed increased cytotoxicity in both of these conditions. In contrast, overexpression of WT CDK5 has no obvious effect on cytotoxicity. This again likely relates to the fact that CDK5 kinase activity requires binding a coactivator such as p35 (10). If expression of p35 or another coactivator is not concurrently increased, then additional expression of WT CDK5 should not be expected to result in any increase in CDK5 kinase activity. When we did overexpress p35 in NK92 cells, their cytotoxicity was reduced. Thus, binding of p35 with CDK5 might be the rate-limiting step for kinase activation in NK cells.

While WT CDK5 overexpression does not appear to have any obvious effect on NK-cell phenotype, including proliferation or cell cycle, CDK5 knockdown in NK92 cells results in cell apoptosis. As p35 knockdown/knockout did not cause this phenotype, and as we did not detect p39 expression in NK cells, this suggests a role for CDK5 that is independent of p35 or p39. Such a role is not unprecedented; for example, in HeLa cells, CDK5 was found to promote the cell cycle, and this role was dependent on the newly identified coactivators CYCLIN I (CCNI) and CYCLIN I-like (CCNI2; refs. 12, 13). CDK5 has known antiapoptotic roles in thyroid and prostate cancer cells and is known to facilitate the DNA damage repair response, and although CDK5 kinase activity in non-neuronal cells is generally described as p35-dependent, it is possible that other, less studied or unknown coactivators enable CDK5 to continue serving these roles even when p35 expression is ablated (39). As an example of the complexity surrounding CDK5 and the cell cycle, in neurons, CDK5 inhibits cell-cycle progression rather than promotes it in a kinase-independent but p35-dependent manner (40). In gastric cancer, CDK5 levels are generally significantly decreased, and CDK5 accumulation in the nucleus actually suppresses proliferation (41). Thus, future research must be conducted with 1° NK cells using improved gene delivery methods to better address the question of whether and how CDK5 knockdown impairs NK-cell survival.

Intriguingly, another member of the CDK family of kinases, CDK8, is reported to play a role in NK-cell cytotoxic function (42, 43). mNK cells isolated from CDK8-deficient *Cdk8^fl/fl^Ncr1Cre* mice exhibited increased cytotoxicity against YAC-1 cells *in vitro*, while *Cdk8^fl/fl^Ncr1Cre* mice had longer survival in a chronic leukemia model compared with control (42). This is consistent with our data showing increased NK-cell cytotoxicity in NK cells from p35 k/o mice. Another similarity to our findings is that *Cdk8^fl/fl^Ncr1Cre* mice did not show any difference in NK-cell count in spleen or bone marrow and did not show changes in CD11b/CD27 maturation compared with the *Cdk8^fl/fl^* control, as we observed in p35 k/o mice (42). The proposed mechanism links CDK8 to the phosphorylation of STAT1-S727 (43). While STAT1-S727 phosphorylation was strongly reduced by CDK8 knockdown and by the pan-CDK inhibitor flavopiridol, the commonly used, nonspecific CDK5 inhibitor roscovitine had no effect, conclusively showing that STAT1-S727 is not a substrate of CDK5.

TGFβ is known to downregulate NK-cell cytotoxicity. In our results, we show an increase in p35 expression in NK cells exposed to TGFβ, while inhibition of TGFβ signaling using EW-7197, a TGFβ receptor inhibitor, reversed this increase in p35. It is interesting to note how quickly TGFβ induces p35 expression in NK cells. Induction of p35 occurs within 2 hours and reaches its highest levels between 4 and 8 hours following the addition of 5 ng/mL TGFβ to 1° NK cells. For comparison, Liang and colleagues observed in MCF-10A breast cancer cells that *p35* mRNA expression was only 2-fold higher at 24 hours and 4-fold higher at 48 hours after treatment with 5 ng/mL TGFβ (22). In addition, the authors noted significant increases in CDK5 expression with TGFβ treatment, in breast cancer cells, while we did not observe such an effect in NK cells. This suggests that regulation of CDK5/p35 expression and kinetics differ in different cells. Our data seem to show that the cytotoxic function of NK cells depends much more on the levels of p35 protein than it does on CDK5. Because IL2 and TGFβ have immune activating and suppressing roles for NK cells, respectively, perhaps the late-stage induction of p35 after IL2 treatment reflects negative feedback signaling meant to return the NK cell to a less active state.

We observed that p25 expression is oftentimes higher than p35 expression in NK cells (Fig. 1C and E), even when p35 is overexpressed (Fig. 4A). Also, while p35 knockdown resulted in loss of the p35 signal, a strong p25 band remained (Fig. 2B). This is likely due at least in part to the greater stability of p25 protein compared with p35, which has a much shorter half-life (9). In addition, whereas p35 levels increase upon IL2 stimulation, levels of p25 remain significantly higher in NK cells (Figs. 1E and 5E). On the other hand, TGFβ increases levels of p35 without apparent increases in p25 (Fig. 5B, D and E). These observations point to a potential mechanistic difference between the impact of IL2 versus TGFβ on the dynamics between CDK5, p35, and p25 in NK cells. p25 could serve a more relevant role in IL2 signaling, and its persistent expression in NK cells could relate to the dependence of NK cells on IL2 for survival in cell culture media *in vitro*. It is also known that p25 lacks the myristoyl signal motif present on the amino-terminus of p35, which targets proteins to cell membranes and affects their intracellular localization (8). This may contribute to distinct roles for p35 and p25 in NK-cell function that should be further explored.

We also observed that shp35 and CDK5-K33T NK92 cells resisted the decrease in cytotoxicity mediated by TGFβ, compared with their respective controls, while OE p35 NK92 cells already exhibited low cytotoxicity and experienced no further decline in cytotoxicity with the addition of TGFβ. We believe that, because p35 is already being overexpressed, TGFβ induction of p35 does not meaningfully contribute any further to inhibition of cytotoxicity. These findings strongly suggest that CDK5/p35 acts downstream of TGFβ signaling in NK cells and plays a role in TGFβ-mediated suppression of NK-cell cytotoxicity. However, the TGFβ signaling pathway is a wide network, and CDK5/p35 most likely regulates only one small portion of it; besides the SMAD-dependent pathway, TGFβ signaling also involves the MAPK, PI3K-Akt, and mTOR pathways, among others (27). The mechanism of how CDK5/p35 negatively regulates NK-cell cytotoxicity and pinpointing where TGFβ signaling intersects with CDK5/p35 activity needs to be further elucidated.

CDK5 inhibitors are known to exhibit anticancer activity, but most of them, including the commonly-used roscovitine, are not specific to CDK5 as they inhibit other CDK proteins such as CDK2, which have well-established roles in the cell cycle (44). Through a structure-guided approach, Khair and colleagues reported a small-molecule inhibitor of CDK5 that is more selective for CDK5 than CDK2 while exhibiting anticancer properties, albeit with lower potency than another, less selective compound (45). We now approach CDK5 and cancer from an immunotherapy angle as well, by which inhibition of the CDK5/p35 would enhance NK-cell antitumor cytotoxicity and inhibit tumor microenvironmental TGFβ-mediated NK-cell dysfunction. Further optimization of a CDK5/p35 kinase inhibitor that is both potent and selective would thus treat cancer through both NK-dependent and NK-independent mechanisms, potentially improving outcomes compared with using just either strategy alone. Alternatively, a gene editing approach may be considered, in which NK cells collected from a healthy donor are modified to no longer express p35, then injected into the cancer patient with the expectation that they display increased resistance to TGFβ-mediated dysfunction and heightened cytotoxic activity against the cancer cells. As NK cells do not display GVHD, interest in NK-cell adoptive therapy and chimeric antigen receptor–NK therapy has continued to increase as strategies to enhance NK-cell cytotoxicity and persistence have evolved and improved (46). Combining these evolving approaches with p35 k/o and/or a selective and potent CDK5/p35 inhibitor could greatly amplify their overall efficacy and should be considered in future translational research.
